# Possible relationship between long-term post neutering complications in dogs and caregiver burden in the owners

**DOI:** 10.3389/fvets.2025.1532039

**Published:** 2025-01-17

**Authors:** Idil Bastan

**Affiliations:** Department of Internal Medicine, Faculty of Veterinary Medicine, Ankara University, Ankara, Türkiye

**Keywords:** gonadectomy, neutering, dog, neutering risks, caregiver burden

## Abstract

Gonadectomy, commonly known as neutering, is widely used to address dog overpopulation and reduce reproductive disease risks, such as mammary cancer and pyometra. It is also advocated for behavior modification. However, neutering is associated with an increased risk of certain musculoskeletal disorders, obesity, several types of cancer, immune-mediated diseases, and cognitive dysfunction. These conditions may necessitate ongoing care and treatment, that require special care that the caregiver must provide furthermore burdens the caregiver with daily tasks, which encompasses the emotional, physical, social, and financial impact on pet owners caring for chronically ill animals. This burden can result in burnout, health issues, depression, social isolation, and financial stress. The potential benefits and risk of gonadectomy can affect the quality of life of both humans and pets. Relevant research findings should therefore be incorporated into each pet's and owner's particular situation. The purpose of this review is to address the long-term neutering risks and the potential caregiver burden that arises from these risks.

## 1 Introduction

Gonadectomy, or more generally referred to as “neutering,” has become a common tool for controlling reproduction and eliminating the risk of reproductive diseases in dogs such as mammary gland cancer, pyometra and prostate hyperplasia/infection, testicular tumors (1–6). Additionally, neutering of dogs and is promoted for population control and behavior modification (1). On the other hand, numerous recent publications have been shown that gonadectomy increases the risk of musculoskeletal degenerative diseases (e.g., hip dysplasia, cranial cruciate rupture), obesity and associated diseases, several forms of cancer, some immune-mediated diseases, urinary incontinence and cognitive dysfunction syndrome (3, 4, 7–11). All these diseases associated with gonadectomy may require ongoing care and treatment, intermittent monitoring and protection. Caregiver burden is defined as the impact on the emotional health, physical health, social life, and the financial status of the pet owner as a result of adopting the caregiver role (12) which has recently been investigated in pet owners caring for chronically ill pets (13–15). Caregiver stress can lead to burnout, acute and chronic bodily illnesses, depression, social isolation, and financial worries, according to studies on owners of chronically ill companion animals (13–15).

The necessity of gonadectomy in dogs remains controversial. This controversy arises primarily from the fact that gonadectomy confers a mixture of benefits and adverse effects that vary depending on the age at neutering, sex and breed (4). Long-term neutering complications can impact not only dog's wellbeing but also the quality of life for the owner. Therefore, the potential long-term health risks related to routine gonadectomy may no longer be acceptable for all pet owners (16).

This review aims to consolidate and present information clearly and concisely, focusing on the possible risks of gonadectomy. Additionally, the review aims to identify possible long-term post neutering complications encountered by the owners of dogs.

### 1.1 Obesity

The owners' perception of their dog's weight is one of their primary concerns regarding neutering (17). Epidemiological studies have shown that neutered dogs of both sexes are more likely to be overweight or obese than intact dogs (18–21). Gonadectomy contributes to obesity through two primary pathways: increased appetite and a lowered metabolic rate. Other explanations for the effect of neutering on obesity include an alteration in feeding behavior leading to increased food intake (22–28) and decreased activity without a corresponding decrease in energy (27). A study found that overweight intact dogs of both sexes lost significantly more weight during the study period than overweight neutered dogs (28).

Overweight and obesity are major risk factors for a number of chronic diseases, including degenerative orthopedic disease (28), diabetes mellitus, insulin-resistance (29–31), urinary tract disease (29–31), skin disease (29, 30), liver dysfunction (32) and neoplasia (33). Obesity not only causes health problems in small pets, but also creates an emotional burden for pet owners (33, 34). Additionally, overweight and obesity also pose a significant financial burden on pet owners. According to a recent report, the owners of overweight dogs spent 17% more on health care and 25% more on medications compared with the owners of healthy-weight dogs (35).

The most important and critical components in reducing the risk of overweight after neutering include monitoring the pet's body weight, making necessary adjustments to the amount and type of food given, and the exercise routine (36). The biggest challenges with a weight-loss program are avoiding overfeeding and gradually cutting back on food intake (37).

Pet obesity have been shown to affect the quality of life in both humans and pets (20). It is the veterinarian's responsibility to discuss the risks of obesity with owners before neutering and to prescribe individual weight maintenance plans after neutering (36, 37).

### 1.2 Skeletal system

Sex hormones play an important role in regulating the elongation of the bones. In dogs that have undergone prepubertal gonadectomy (before 6 months of age), growth plate closure is delayed (38). Many studies have reported that neutered dogs have a significantly higher risk of developing orthopedic disorders than intact dogs. The most common orthopedic problems are cranial cruciate ligament rupture, hip dysplasia, elbow dysplasia, and osteoarthritis (39–41). The relationship between neutering and osteoarthritis is further complicated by the fact that neutered dogs are more likely to be obese (42).

Clinical signs associated with canine orthopedic disorders include limping or lameness, exercise intolerance, pain, inactivity stiffness, reduced willingness to jump up and/or down, willingness to play, aggression, vocalization, and postural shifting (43). Dogs with orthopedic diseases experience barriers such as difficulties with daily activities, which can make them dependent on their owners and dramatically compromise their welfare. Caring for dogs suffered from orthopedic diseases could be physically and emotionally demanding tasks for their caregivers. The multifaceted negative impacts of owning an osteoarthritic dog include: the stress of veterinary visits; feeling they had let their dog down by failing to find effective treatments; challenges of getting the dog to take medications; and keeping the dog at a reasonable weight and body condition score whilst on a restricted exercise regime. As a result of their pets' orthopedic conditions many dog owners have experience undesirable challenges such as financial difficulties due to treatment costs; quitting their jobs in order to take care of their dogs; changing work schedules, room layouts, vehicles and furniture; back pain from carrying or assisting big dogs up and down stairs or helping them in and out of the car (44).

### 1.3 Neoplasia

Neoplasia, a major cause of morbidity and mortality for pets, is one of the leading causes of concern among pet owners (45, 46). Associations have been made between the development of some types of neoplasms and neuter status as well as genetic predisposition, age, viral infection, chronic inflammation, and breed; however, the etiology is likely multifactorial and largely unknown (45–48).

Gonadectomy has the potential to decrease the incidence of certain types of neoplasia like mammary tumors, ovarian tumors, uterine neoplasms, vaginal and vulvar tumors in female (4, 5, 45–52); testicular tumors in males (53, 54). On the other hand, gonadectomy may be associated with increased risk of development of certain neoplasms such as canine prostate cancer, lymphoma, mast cell tumors, hemangiosarcoma and osteosarcoma (3, 11, 55–64).

Mammary tumors are the most frequent neoplasia in female dogs (4, 52). Delayed spaying increases the risk of developing mammary tumors (54–57). Intact female dogs are at four times greater risk of developing mammary tumors, compared to those spayed before 2 years of age (5). The incidence of mammary tumors in cats is less than half that of dogs (53). According to a study, the protective effect of spaying at an early age, displaying a 91% and 86% reduction in development of mammary tumors in cats spayed before 6 months and 7–12 months, respectively (57).

One of the most often diagnosed canine neoplasia is lymphoma (45–47). In dogs, the reported incidence of lymphoma is 1.1% (64). A comparative medicine study showed that sexually intact male dogs and neutered male and female dogs had twice the risk of developing lymphoma compared to sexually intact female dogs (65). In Golden Retrievers, neutered males were three times more likely to develop lymphoma than unaltered male. However, there was not a significant risk of developing lymphoma associated with spaying females at any age (56). Another study on Vizslas, analysis suggested that both neutered males and females were 4.3 times as likely as their sexually intact counterparts to have lymphoma. In addition, the study revealed that the odds of gonadectomized Vizslas of either sex developing mast cell tumors were 3.5 times the odds of sexually intact Vizslas developing mast cell tumors (55). However, there was not a significant difference between the incidence of mast cell tumors in males and females regardless of neuter status or time of gonadectomy in German Shepherd Dogs (57).

Neutering is associated with risk of developing splenic and cardiac hemangiosarcoma, in particular. Compared to dogs that were sexually intact, gonadectomized females younger than 12 months and gonadectomized males and females older than 12 months had a markedly higher risk of developing hemangiosarcoma (62). It has been found that spayed females have a higher risk of developing splenic and cardiac hemangioma compared to intact females (58, 66).

The most prevalent primary malignant bone tumor in dogs is osteosarcoma (49, 59, 60). Incidence of canine osteosarcoma is < 0.1% (67). Male and female Rottweilers underwent gonadectomy before the 1 year of age had an approximate one in four-lifetime risk for bone sarcoma and were significantly more likely to develop bone sarcoma than dogs that were sexually intact (61). Neuter status or age at gonadectomy does not affect development of osteosarcoma in German Shepherds (57).

Canine prostate cancers are rare (< 0.6% in necropsy studies) (68), but it is often aggressive, rapidly proliferative, and highly metastatic to the skeletal system (69). Although castration has both preventive and therapeutic effects on androgen-dependent diseases such as benign prostatic hyperplasia chronic prostatit and perineal adenomas (70–72). Several studies have shown that castrated male dogs exhibit an elevated risk of developing prostate cancer, with odds ratios ranging from 3.6 to 4.34 and experience more metastases than intact dogs (3, 11, 73–76).

Pet owners are particularly concerned about cancer since it is a major cause of morbidity and fatalities in pets (45, 46). Cancer is an emotionally charged disease (62). The burden of caregiving for a companion animal with cancer can be distressing for the owner. Recent studies indicate that pet owners experience depressive symptoms after their pet gets diagnosed with cancer (63). Silva et al. found that a significant portion of guardians of ailing animals with oncological pathologies, irrespective of their pets' conditions, faced substantial burdens, impacting their emotional, physical, and financial wellbeing (17).

### 1.4 Behavior

Behavioral problems are an important reason for relinquishment of pets by owners (77, 78). The most common behavioral problems of dogs include aggression toward people or other animals, inappropriate elimination, and fearful behavior (79). Neutering has a complex effect on behavior, with both beneficial and detrimental consequences documented (80, 81). Non-sexually dimorphic behaviors, such as fear-based aggression, are not affected by gonadectomy (81). Gonadectomy reduces reproductive behaviors by eliminating estrus-related actions in females and reducing roaming, mounting and urine marking in male dogs (82–84).

Although neutering is generally thought to reduce the incidence of various types of aggression, there is some evidence that it may increase some aggressive behaviors. Several studies of dogs referred for treatment of behavior problems have identified a higher proportion of spayed than intact females among animals exhibiting aggression (85, 86). Early age gonadectomy has been reported to increase noise phobia and decrease separation-related disorders, escape behaviors, and elimination when frightened (83, 87). Male and female neutered dogs are more prone to exhibit symptoms of obsessive-compulsive disorder (88, 89), stranger-directed aggression (90, 91).

Behavioral problems are a prevalent cause of the disruption of the human-animal bond, jeopardizing their mutual wellbeing and may result in pet abandonment and euthanasia (78, 92, 93).

The owners of pets with behavioral problems often feel isolated and judged due to the lack of understanding and unsupportive reactions of the trainers, veterinarians, strangers, friends, and family members (93). In order to improve the welfare of both owners and pets, practical behavioral support for the dog-human dyad, social and psychological support for owners should be provided (94). Many behavioral disorders require complex multimodal therapy including environmental management, behavior modification, changes in owner behavior and routines, and medication administration; thus, the emotional and financial efforts committed to managing a pet with behavioral disorders would be expected to lead to burdening the caregiver (93–95). Owners of pet with behavioral disorders face many challenges in their daily lives. These challenges include the extra time required for management and training, the difficulties in handling the pet in public and when alone at home, the level of planning and attention required to keep their pet and others safe, the cost of behavioral support for their pet, and how the problem limits the owner's time away from home (78).

### 1.5 Urinary system

In intact female dogs, the risk of urine incontinence (UI) is low (96). Approximately 75% of female dogs become incontinent within 3 years after gonadectomy, whereas UI following spaying can occur right away or up to 10 years after gonadectomy (83). Several variables, including body weight, breed, and timing of neutering, contribute to the risk of developing UI (97). O'Neill et al. reported that urinary incontinence affects just over 3% of female dogs overall but affects more than 15% of female dogs in high-risk breeds including the Irish setter, Dobermann, Bearded Collie, Rough Collie and Dalmatian (97).

Urinary incontinence is a life-long condition that requires continuous medication. UI can be distressing and frustrating for the owner and may negatively impact the interactions between owners and their pets (98). A total of 10%−20% of affected families have reported unfavorable outcomes of UI, with owners describing feelings of anger and discomfort (99) and with euthanasia of the affected animal considered in certain circumstances (100). Long-term soiling of the house due to urinary incontinence can be exasperating and put strain on the owner-pet bond, as well as requiring the investment of time and money in cleaning, repairing and sometimes even replacing soiled possessions (101).

### 1.6 Other disease risks

Sundburg et al. evaluated the potential association between neutering and diseases associated with immune function compromise in dogs. The authors concluded that neutered dogs had a significantly greater risk of hypoadrenocorticism, autoimmune hemolytic anemia, atopic dermatitis, hypothyroidism, inflammatory bowel disease and thrombocytopenia than intact dogs with neutered females being at greater risk than neutered males for all but hypoadrenocorticism and autoimmune hemolytic anemia. Neutered females, but not males, had a significantly greater risk of Lupus erythematosus than intact females (102). Diabetes mellitus has been reported to be more common in neutered males, but not female dogs compared to intact dogs across the whole dog population (103).

A chronic disease is a condition requiring lifelong support, protection, intermittent monitoring, and continuous treatment (104). Caregiver burden is related to treatment complexity, and notably the relationship to treatment complexity is not related to the diseases' severity. Chronic diseases like atopic dermatitis (105), inflammatory bowel disease (106), and hypoadrenocorticism (107) in a companion animal causes burden for the owner. Chronic illness also has an impact on the owners' social life, daily activities, job, finances, leisure time, travel, and family relationships (105, 108, 109).

## 2 Conclusions

Decision-making in gonadectomy remains controversial. It is apparent that gonadectomy has both benefits and risks for individual dogs. Given the multitude of interacting etiological factors that play a role in most serious behavioral and medical conditions, it is impossible to predict the exact outcome of neutering for any individual. Any decision to neuter a particular pet should be made considering individual circumstances, the owners' values and goals, as well as the risks and benefits determined by epidemiological data.

Veterinarians should consider possibility of caregiver burden that affects family relationships, social life, daily activities, work, finances, leisure time, and travel of the owner when recommending neutering. They should also discuss with their clients about the potential long-term complications associated with neutering and the burdens that the owners may face.
